# Our “Society Reports”

**Published:** 1894-09

**Authors:** 


### OUR "SOCIETY REPORTS."

We wish to make it a special feature of THE JOURNAL in the future to publish full and correct reports of the meetings of medical societies in this State. We have several good societies at work, and heretofore the transactions of some of them have only been recorded by the Secretary. We have published, from time to time, the proceedings of the Atlanta Society of Medicine and of the Macon Medical Society. We call attention this month to a report of the former on "Conservatism in Abdominal and Pelvic Surgery." Next month we will report the Augusta Academy of Medicine and the Atlanta Obstetrical Society. Other societies in the State will also be reported when possible. We think this feature of THE JOURNAL will be of interest to our readers and in the interest of the State profession.

